# COVID-19 Vaccine Status, Intent, Hesitancy, and Disease-Related Beliefs in People with Multiple Sclerosis

**DOI:** 10.3390/vaccines11020410

**Published:** 2023-02-10

**Authors:** Lisa Grech, Alastair Kwok, Mike Nguyen, Antony Winkel, Ernest Butler, Michelle Allan, Nathan Bain, Eva Segelov, on behalf of the MSVACCS Investigators

**Affiliations:** 1 Department of Medicine, School of Clinical Sciences, Monash University, Clayton, VIC 3168, Australia; 2 Department of Oncology, Monash Health, Clayton, VIC 3168, Australia; 3 Department of Neurology, Sunshine Coast Hospital and Health Service, Birtinya, QLD 4575, Australia; 4 School of Medicine, Griffith University, Birtinya, QLD 4575, Australia; 5 Monash Neurology, Monash Health, Clayton, VIC 3168, Australia; 6 Department of Clinical Research, Faculty of Medicine, University of Bern, 3012 Bern, Switzerland

**Keywords:** COVID-19, vaccine hesitancy, vaccine intent, disease-related beliefs, multiple sclerosis

## Abstract

Background: People with multiple sclerosis (MS) are susceptible to severe COVID-19 outcomes. They were included as a priority group for the Australian COVID-19 vaccine roll-out in early 2021. However, vaccine hesitancy remains a complex barrier to vaccination in this population group, which may be partly related to disease relapse concerns following COVID-19 vaccination. This study examined the COVID-19 vaccination status, intent, hesitancy, and disease-related beliefs in people with MS. Methods: An online survey was conducted with people with MS receiving care at two Australian health services between September and October 2021. It collected sociodemographic and disease-specific characteristics and responses to validated scales that assessed vaccine hesitancy and general and MS-related vaccine beliefs. Results: Of the 281 participants [mean age 47.7 (SD 12.8) years; 75.8% females], most (82.9%) had received at least one COVID-19 vaccine dose. Younger participants were less likely to be vaccinated, as were those within 1–5 years of disease duration. After controlling for age, disease duration was not associated with vaccination status. Unvaccinated participants were more likely to report less willingness to receive the COVID-19 vaccine, higher vaccine complacency and lower vaccine confidence, greater MS-related vaccine complacency, and higher MS and treatment interaction concerns. Conclusions: People with MS reported a high vaccination rate, despite general and MS-specific COVID-19 vaccine concerns. Greater MS-specific concerns were reported by those who indicated that their MS was not well-controlled and their MS impacted their daily activities. By understanding the factors that influence vaccine hesitancy and their interplay with MS disease course and treatment concerns, this can inform tailored interventions and educational messages to address these concerns in people with MS. Clinicians, governments, and community organisations are key partners in delivering these interventions and messages, as ongoing booster doses are needed for this vulnerable population.

## 1. Introduction

Multiple sclerosis (MS) is a neurodegenerative autoimmune disease affecting 2.8 million people worldwide, with severe and disabling consequences [1]. While people with MS contract severe acute respiratory syndrome coronavirus 2 (SARS-CoV-2) at similar rates to the general population, they are at greater risk of severe COVID-19 outcomes, including long-COVID [2,3]. In addition to MS-specific risk factors, the higher rate of comorbidities, including depression, hypertension, hypercholesterolaemia and chronic lung disease, potentially compounds the risk of poor COVID-19 outcomes [4].

COVID-19 vaccination is a key strategy for limiting the health and social consequences of SARS-CoV-2, including reducing disease severity. Consistent with increased vulnerability, people with MS were prioritised for COVID-19 vaccinations across many countries [5,6] and have been recommended for extended vaccination schedules (5 vaccines in total) [7,8]. Despite this, studies have identified vaccine hesitancy concerns in people with MS [9,10,11]. 

Vaccine hesitancy is a complex and context-specific phenomenon that refers to a delay or refusal of vaccine uptake in the presence of available vaccination services [12]. Even prior to the COVID-19 pandemic, it was listed by the World Health Organisation as one of the top 10 global health threats [13]. 

COVID-19 vaccine hesitancy has been well described in general population studies to be associated with factors including efficacy, safety, side effects, convenience and price, and varies considerably across populations, countries, and timepoints [14]. Beliefs associated with hesitancy included insufficient vaccine testing, authorities motivated by financial gain, which the health risk of COVID-19 had been exaggerated and that natural exposure to the virus provided the safest protection. Socio-demographics associated with higher levels of hesitancy included being female, being younger in age, having a lower income, and having a lower education [14].

For people with MS, vaccine hesitancy may be related to concerns about the effect of vaccination on their disease and/or disease-related treatments. Concerns about the ability of vaccinations to trigger autoimmune diseases generally, and in MS specifically, have been identified [15]. While a causal link is inconclusive, there have also been case reports about people with MS who incurred disease relapse shortly following the COVID-19 vaccine administration [16]. Given this, it is unsurprising that COVID-19 vaccine hesitancy has been associated with anxieties about the interplay of the vaccine with both the disease progression and treatment [10].

Previously validated hesitancy scales were developed for the general population without consideration of disease or treatment-specific concerns. While people with MS experience many of the same hesitancy concerns, it is important to understand the unique contribution of disease-specific concerns in vaccination decision making. We developed and validated a six-item scale, which has two factors that address concerns related to COVID-19 vulnerability due to underlying disease and concerns related to efficacy, safety and impact on MS treatments and disease course [17]. 

The aims of this study were to identify the following: (1) demographic and disease-specific contributing factors that may influence COVID-19 vaccination decision-making in people with MS; (2) the ability for a disease-specific vaccine hesitancy scale to determine vaccination status; (3) whether disease-specific vaccination factors contributed beyond that of general population vaccination hesitancy beliefs and concerns.

## 2. Materials and Methods

### 2.1. Study Design

**Figure 1:**
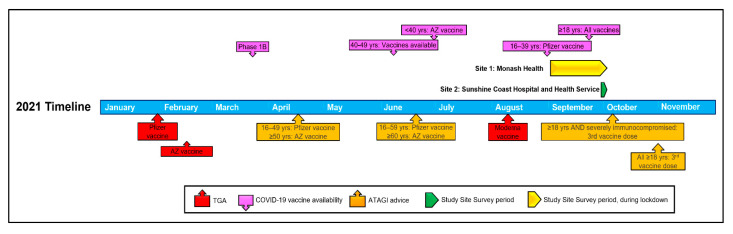
Timeline of the survey period with notable Australian COVID-19 vaccine roll-out events in 2021. People with MS could access COVID-19 vaccination from the commencement of Phase 1B during the COVID-19 vaccine rollout in March 2021. The survey period at Monash Health was undertaken during a strict state-wide government lockdown. Abbreviations: ATAGI, Australian Technical Advisory Group on Immunisation; Years, Yrs; AZ, Astra-Zeneca; TGA, Therapeutic Goods Administration—COVID-19 Vaccine Provisional Registration.

This cross-sectional study was implemented at two public health services across two states in Australia, encompassing one metropolitan and one regional/rural area. The survey was open from 2 September to 5 October 2021. There were public health restrictions and vaccine rollout recommendations in effect during the survey period (Figure 1). The study was approved by the Monash Health Human Research Ethics Committee (RES-21-0000-364L-76466) and registered with the Australian New Zealand Clinical Trials Registry (ACTRN12621001467820).

### 2.2. Study Participants

Participants were eligible if they had a past or current diagnosis of MS, were aged 18 years and over, and a patient of either of the two participating health services. Potential participants were invited by text message with a link to the study information and consent if they had attended a neurology appointment within the past 12 months. A reminder text message was sent following the initial invitation.

Potential participants who accessed the survey link and provided written informed consent were directed to the survey, hosted on the Qualtrics^®^ secure data capture platform. Paper surveys were available upon request. The survey was presented in English and was completed anonymously. Caregivers and family members were encouraged to assist participants with completing the survey.

### 2.3. Measures

The 44-item survey was developed by a panel of clinicians, researchers, and patient representatives (Table S1). It took approximately 10–15 minutes to complete. All scale items used a 5-point Likert scale plus a ‘don’t know’ option. No identifiable information was collected. 

**Vaccine uptake status, demographics, and clinical history:** Demographic factors were collected, including gender, age, highest level of education, range of annual household income, and whether English was the participant’s first language. Clinical history items included disease type, time since diagnosis (within a range), current treatment, MS control, disease-modifying therapy (DMT) adherence, and impact of disease on daily activities.

**Oxford COVID-19 Vaccine Hesitancy Scale (OCVHS):** This seven-item scale assesses willingness to receive a COVID-19 vaccine, with higher scores indicating greater vaccine hesitancy [18].

**Oxford COVID-19 Vaccine Confidence and Complacency Scale (OCVCCS):** This 14-item scale measures COVID-19 vaccine confidence and complacency attitudes and comprises a summary scale and the following four subscales measuring: collective importance of a vaccine, belief that COVID-19 infection may occur, and the vaccine will work, speed of vaccine development, and side-effects [18]. Higher scores indicate greater negative attitudes toward vaccination.

**Disease Influenced Vaccine Acceptance Scale-Six (DIVAS-6):** This six-item scale assesses COVID-19 vaccine attitudes related to disease and treatment. It is comprised of two subscales, disease complacency and vaccine vulnerability. A higher score for the disease complacency subscale indicates greater disease-related vaccine complacency, whereas a higher score for the vaccine vulnerability subscale indicates a higher level of perceived disease- and treatment-related vaccine concerns [17].

**Disease impact on lifestyle:** One item was developed to measure the impact of MS on daily activities in the past four weeks. Response choices ranged from ‘Not at all’ to ‘All the time’.

### 2.4. Statistical Analysis

Unsubmitted incomplete, duplicate, and ineligible survey responses were removed prior to analysis. Imputation was not used for missing data. Summary scores were calculated for each scale and subscale for the OCVCCS and the DIVAS-6. ‘Don’t know’ responses were not scored or analysed, consistent with previous approaches [18].

Descriptive statistics summarized the survey data, including socio-demographic and clinical characteristics and scale and subscale scores. Due to a low number of responses, the categories of some socio-demographic variables were either combined or removed for analysis as follows: (1) no formal education level, primary education level and secondary education level were combined for the highest level of education variable; (2) non-binary/other gender were removed for analysis.

Cross-tabulation between categorical variables, including demographics and individual scale items (item five ‘intent to vaccinate’, item 12 ‘likelihood of COVID-19 infection’ and item 28 ‘doctor’s recommendation’) were analysed using chi-squared tests. Logistic regression was used to determine whether the demographic, disease-related variables, and scales (summary score, subscale score and items) predicted vaccination status. Linear regression evaluated whether demographic and disease-related predicted scale scores. Demographic and disease-related variables were entered into time-controlled hierarchical regression analyses if they were significantly correlated with the outcome variable (i.e., a correlation coefficient between −1 and +1, using Pearson’s and Spearman’s Rho). The *p*-values < 0.05 were considered significant. Effect sizes were calculated with the phi coefficient (φ) and Cramér’s V (φ*_c_*) for chi-squared tests, and eta squared (η^2^.) for independent sample *t*-tests. Receiver operating analysis was conducted to evaluate the diagnostic ability of the OCVHS, the OCVCCS, and the DIVAS-6 in discriminating the participant’s self-reported vaccination status. (“vaccinated” v. “unvaccinated”) as the gold standard, Cases were classified as “vaccinated” and “not-vaccinated” as the gold standard, with the former indicating a positive case for sensitivity, whereas the latter corresponding to a negative case for specificity. Statistical analyses were performed using SPSS Version 27.0 (IBM, Chicago, IL, USA). Findings from this study were reported according to STROBE (strengthening the reporting of observational studies in epidemiology) guidelines [19].

## 3. Results

### 3.1. Participant Characteristics

Of the 997 delivered text messages, there were 432 survey responses, of which there were 281 eligible responses for analysis. Ineligible responses comprised 112 incomplete and three duplicate responses, resulting in a survey response rate of 31.8% (317 of 997). A further 36 ineligible responses were removed for reasons of <18 years of age (n = 3), not diagnosed with MS (n = 29), and not receiving care at a participating site (n = 4).

Socio-demographic and clinical characteristics are detailed in Table 1. Consistent with MS gender distribution, there were more female participants. The mean age (SD) was 47.7 (12.8) years. English was the dominant language (>90%), and 85% attended a metropolitan-based health service. Types of MS were as follows: 72% relapsing-remitting, 11% secondary progressive, and 9% primary progressive MS, with 8% responding “other/don’t know”. Most participants (79.7%) were on current DMT. 

### 3.2. Vaccination Status and Intent

Overall, 233 participants (82.9%) reported receiving at least one COVID-19 vaccine at the time of the survey. Comparing vaccinated and unvaccinated participants, the latter were younger and more likely to have been diagnosed within the past 1–5 years (Table 2). On multivariable regression, age remained a significant association (Table S2).

With regards to accepting COVID-19 vaccination amongst the 48 unvaccinated participants, 21 stated that they were likely to, 13 that they were unsure, and 14 that they were unlikely to undergo vaccination. There was a significant relationship between intention to get vaccinated and whether their doctor’s recommendation was important (*X*^2^ [*df* 4] =13.22, *p* = 0.01). There was no difference between vaccinated and unvaccinated participants in relation to their belief about whether they were likely to contract COVID-19 within the next 12 months (B(SE) 0.16 (0.15), *p* = 0.27).

### 3.3. Vaccine Hesitancy, Confidence and Complacency

Participants who were unvaccinated reported significantly higher OCVHS scores compared to vaccinated participants. Similarly, unvaccinated participants reported significantly higher scores on the OCVCCS total indices and each of the sub-indices, indicating higher concerns about the speed of development and side effects and more negative beliefs regarding collective importance and potential therapeutic benefits (Table S3).

**Demographic factors:** Being female and of younger age was significantly related to higher OCVHS scores (Table 3). Younger age was also associated with higher summary and subscale scores measuring negative beliefs about the vaccine and concerns about vaccine side effects from the OCVCCS (Table S4). Participants in metropolitan locations reported lower OCVCCS summary scores, lower levels of vaccination collective importance, and lower concerns about the speed of development than participants in regional/rural locations. 

**Disease-specific factors:** When compared with people with a diagnosis of MS longer than 10 years, those with a diagnosis of MS within 1–5 years reported greater vaccination hesitancy (Table 3), although this did not remain significant when controlling for age and gender (Table S5). People with a diagnosis duration between 1 and 5 years also had more negative attitudes around overall vaccine confidence and complacency, side effects, and vaccine beliefs (Table S4). There were also more negative ‘beliefs’ for people diagnosed between 5 and 10 years on the OCVCCS vaccine beliefs subscale. When age and location were controlled in multiple regression analysis, the OCVCCS summary score remained significantly higher for people who had been diagnosed with MS for between 1 and 5 years compared to those diagnosed for longer than 10 years (Table S6). When age was controlled, negative beliefs about vaccination also remained significantly higher for people diagnosed between 1 and 5 years (Table S7), whereas concerns about side effects were no longer significant (Table S8).

### 3.4. Disease-Related Vaccine Concerns (DIVAS-6)

**Figure 2:**
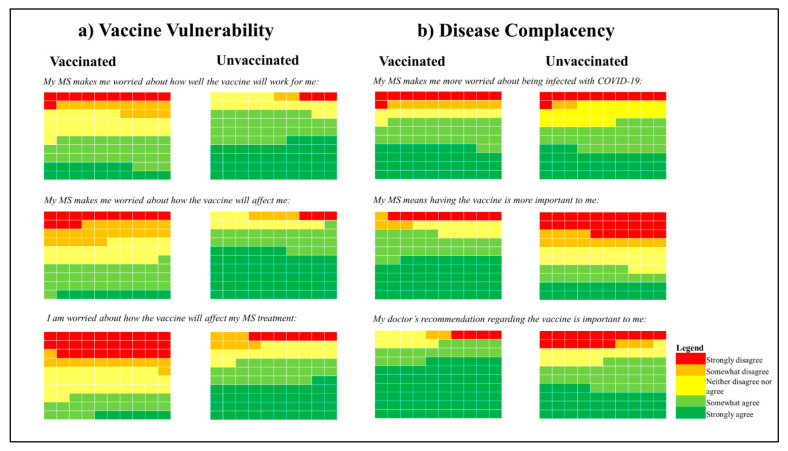
The frequencies of the five possible responses for each item of the DIVAS-6 subscales: (**a**) vaccine vulnerability (**b**) disease complacency. Each single-coloured box represents 1% of patient participant responses for each item, by vaccination status. ‘Don’t know’ responses were excluded. Abbreviations: DIVAS-6, Disease Influenced Vaccine Acceptance Scale-6.

**Response frequencies:** When asked about vaccine concerns in relation to their MS diagnosis, unvaccinated participants reported greater concerns about vaccine efficacy, side effects, and interactions with MS treatment (Figure 2a). Regarding the statement, ‘My history of MS makes me more worried about being infected with COVID-19,’ the proportion who agreed was similar between unvaccinated and vaccinated participants (Figure 2b). The statements ‘My history of MS means having the vaccine is more important to me’ and ‘My doctor’s recommendation regarding the vaccine is important to me’ were more likely to have an agreement by vaccinated participants than unvaccinated participants.

**Demographic factors:** A higher DIVAS-6 summary score was reported by participants who spoke English as a non-dominant language and participants of younger age (Table S9). When both age and English as a first language were entered into multivariable regression, only the latter remained significantly associated with the summary score (Table S10). Greater disease-related vaccine vulnerability was seen among female participants and participants of younger age (Table S9). No demographic factors predicted disease-related vaccine complacency. 

**Disease-specific factors:** When compared with participants diagnosed for 10 years or longer, participants who were diagnosed within 1–5 years reported higher vaccine vulnerability, as did participants who reported that their MS was not well controlled in the previous 6 months, compared to those who reported their MS was well controlled. When gender and age were controlled, disease duration was no longer significant, while MS control remained significant (Table S11). People who reported MS had no impact on their daily life showed significantly lower disease-related vaccine vulnerability than people who reported that MS impacted their daily life ‘all of the time’, ‘some of the time’ or ‘not very often’, while ‘most of the time’ was not significant (Table S9). When relevant demographic variables were controlled in multivariable analysis, ‘most of the time’ also reached significance, such that participants with no impact of MS on daily activities reported lower vaccine vulnerability than participants experiencing any level of MS impact on their daily activities (Table S12).

Participants with primary progressive MS reported significantly higher disease complacency scores compared with those with relapsing-remitting MS (Table S9). No disease-specific factors predicted the DIVAS-6 summary scale score.

### 3.5. Predicting Vaccine Status

Using a dichotomous measure of self-reported vaccination status (Yes = one or two doses, No = no doses) as the gold standard, receiver operating analysis determined the diagnostic utility of the OCVHS, the OCVCCS, and the DIVAS-6. Using Hosmer and Lemeshow’s guidelines [20]. Results showed all summary scores and three of the OCVCCS subscales provided excellent discriminative ability. The OCVCCS Beliefs about COVID-19 subscale provided acceptable discriminability, while the DIVAS-6 disease complacency subscale provided limited discriminability (Table S13). 

A score of 13 provided the best predictive ability for the OCVHS, determined by a Youden’s Index of 0.59. This provided a sensitivity for predicting vaccination of 71.1% and a specificity for predicting the absence of vaccination of 88.1%. For the OCVCCS, a score of 32 provided a sensitivity of 65.0% for being vaccinated and a specificity of 91.6% for being unvaccinated (Youden’s Index: 0.57). There was no Youden’s Index above 0.5 for the DIVAS-6 summary scale, highlighting that there is not a single best cut-off score to maximise the prediction of sensitivity and specificity. A cut-off score of 15 provided a sensitivity of 90.2%, and a score of 20 provided 93.0% specificity. Sensitivity and specificity values for each cut-off score are provided (Tables S14–S22).

## 4. Discussion

This study assessed the COVID-19 vaccination rate, vaccine hesitancy, and beliefs in people with MS, using validated tools, including the DIVAS-6, a tool to assess disease-specific vaccine concerns. Important findings are the increased disease-related vaccine concerns for people who perceived their MS was not well-controlled and for those reporting any impact of MS on daily activities. Vaccine hesitancy and concerns were higher for participants who were unvaccinated and were predicted by the following demographic factors: age, gender, and location, and by the following MS-specific factors: disease duration, level of control and impact on daily activities. Despite people with MS being prioritised for COVID-19 vaccination, the proportion of participants who had received at least one COVID-19 vaccination was similar to the general Australian public at the time of the survey (approximately 82% versus 81%, respectively) [21]. 

People with MS who did not consider their MS well-controlled were less likely to be vaccinated and reported higher results on the disease-related vaccine vulnerability subscale, which measures concerns about the impact of the vaccine on the disease or treatments. Similarly, those who considered that their MS did not impact their daily activities, compared to those that reported any amount of MS-related impact on daily activities, reported lower disease-related vaccine vulnerability. This extends the understanding of the interplay between COVID-19 vaccine concerns and disease stability. It has been previously reported that people with MS have concerns about the impact of the vaccine on disease relapse or progression [9,22,23]. Fear about reduced DMT efficacy and lack of DMT interaction knowledge has also been cited [23,24,25,26]. While one study has reported that participants taking DMT were more likely to be vaccinated than those who were not [27]. On balance, it appears that currently active MS and people with perceived MS impairment do not want to risk an unknown vaccination impact. 

It is unsurprising that people with MS experience concerns about the effect of the vaccine on their disease, given published case reports of the temporal association between COVID-19 vaccination and disease activation [16]. This is on a backdrop of previous concerns about vaccination and disease activation, although they are generally unfounded [28]. We found that compared to people with a diagnosis duration greater than 10 years, those with a duration of 1–5 years reported higher hesitancy, negative beliefs, and side-effect concerns. This was not seen in those diagnosed within 12 months. The first five years of MS disease duration is often considered the ‘newly diagnosed’ stage. It is possible that people in this phase experience more concerns about the vaccine for their disease because they are adjusting to their diagnosis; however, within the first 12 months, the more frequent contact with healthcare providers at this stage may have facilitated education and advice on COVID-19. 

Advice from a healthcare provider has been related to being more accurately informed and having greater intent to get COVID-19 [23,26,29]. In our study, approximately 71% of participants who were not vaccinated reported that they were likely or unsure if they would accept a COVID-19 vaccination. This indicates an opportunity to improve vaccination rates via healthcare professional consultation. Ehde et al. [9] reported that 90% of people with MS who were undecided about whether they would accept COVID-19 vaccination wanted more information to help make their decision. In the current study, 66% of unvaccinated participants considered their doctor’s recommendation about COVID-19 vaccination important, emphasising the need for healthcare collaboration to maximise vaccination and ongoing booster uptake in people with complex chronic conditions such as MS. The importance of shared doctor-patient decision-making is well established [30]. Additionally, people with MS who had discussed COVID-19 vaccination with a healthcare provider reported significantly higher intention of vaccine uptake [26]. This is highlighted in research that found some people with MS delay COVID-19 vaccination until they receive advice from their neurologist [22,23].

While COVID-19 vaccination is recommended for all people with MS [31]. One study reported that 7% of unvaccinated participants stated their doctor advised against getting the COVID-19 vaccination [27]. This highlights the importance of educating and resourcing the multidisciplinary clinicians encountered by people with MS to maximise their contribution as important partners in vaccine campaigns [32]. This strategy works in tandem with the broad information campaigns developed by governments and non-governmental organisations to alleviate general vaccine concerns [33]. 

We found that the vaccine hesitancy, confidence and complacency, and DIVAS-6 summary scales can be used to predict vaccine uptake with excellent discrimination. The DIVAS-6 can be utilised to identify disease-related vaccine concerns that require addressing in vaccinated people and those at risk of not accepting vaccination or boosters using different cut-off scores depending on the purpose. For example, a score of 20 has a specificity (likelihood the person is not vaccinated) of over 90%, which is useful if only wanting to target those who are not vaccinated, whereas if the aim is to identify those who are vaccinated, a score of 15 provides a sensitivity of approximately 90%. Identifying disease-related vaccination concerns using the DIVAS-6 can prompt, inform, and enable targeted healthcare provider conversations. This is useful for patients who are less likely to proactively verbalise barriers to them receiving the vaccination.

Participants of younger age were more likely to be unvaccinated and report greater vaccine hesitancy and disease-specific vaccine concerns, more negative beliefs about vaccination, and greater vaccine side-effect concerns. Younger age, in particular, has been shown to be related to COVID-19 vaccine hesitancy, generally and in MS [10,14,34]. Our research also found females reported significantly higher vaccine hesitancy than males, including disease-specific concerns, while the vaccination rate was not significantly different. Vaccine hesitancy has been shown in general population samples broadly [14]. Disease-related vaccination concerns were more likely in participants reporting English as their non-dominant language. This again points to the need for appropriate healthcare provider consultation and supportive resources that are understandable to people of culturally and linguistically diverse backgrounds [35].

Study limitations included a lower-than-expected prevalence of people with secondary progressive MS [36], as well as recall and misclassification bias inherent in any survey-based study. Our sampling method meant that people with a greater interest in COVID-19 vaccinations, those who were highly educated and live in more metropolitan areas, and those who have access to a computer or mobile phone may have been more likely to respond. Availability only in English was another limiting factor, which may have affected the participation of non-English speaking patients. Our finding that participants had higher disease-related vaccine concerns than those with English as their primary language highlights the need for future research using methods accessible to people from culturally and linguistically diverse backgrounds. Compared to other studies, the relatively low community transmission and high vaccination rates at the time of this study in Australia should be considered. 

## 5. Conclusions

This study highlights the importance of addressing MS-specific concerns related to COVID-19 vaccination, particularly the potential impact on disease activity and/or DMTs. The role of the clinician in addressing vaccine concerns is shown to be key. The findings are of particular relevance to people in the earlier years of diagnosis and with a disease that is not stable. The utility of the Oxford inventories and the DIVAS-6 validated scales to relate vaccination status with disease-related concerns is demonstrated. Clinicians should be encouraged to use these scales to identify and target patient information about COVID-19 vaccination, particularly as the need for boosters and the ongoing threat of COVID-19 evolves.

## Figures and Tables

**Table 1:** Participant characteristics.

Characteristic	Total n = 281 (%)	Vaccinated n = 239 (82.9%)	Unvaccinated n = 48 (17.1%)
Male	65 (23.1)	57 (87.7)	8 (12.3)
Female *	213 (75.8)	174 (81.7)	39 (18.3)
Age: mean (SD)	47.7 (12.8)	49.1 (12.6)	40.6 (11.7)
**Age (years)**			
18–39	83 (29.5)	56 (67.5)	27 (32.5)
40–59	138 (49.1)	120 (87.0)	18 (13.0)
≥60	60 (21.4)	57 (95.0)	3 (5.0)
Highest level of education **			
No formal/primary school	4 (1.4)	3 (75.0)	1 (25.0)
Secondary school	86 (30.6)	73 (84.9)	13 (15.1)
Vocational/Trade	75 (26.7)	57 (76.0)	18 (24.0)
University	115 (40.9)	100 (87.0)	15 (13.0)
**Annual household income (AUD)**			
<50 K	72 (25.6)	62 (86.1)	10 (13.9)
50 K–100 K	76 (27.0)	59 (77.6)	17 (22.4)
100 K–150 K	41 (14.6)	33 (80.5)	8 (19.5)
>150 K	40 (14.2)	35 (87.5)	5 (12.5)
Prefer not to say	52 (18.5)	44 (84.6)	8 (15.4)
**English as first language**			
Yes	257 (91.5)	215 (83.7)	42 (16.3)
Location			
Metropolitan	239 (85.1)	202 (84.5)	37 (15.5)
Regional/Rural	42 (14.9)	31 (73.8)	11 (26.2)
**Multiple sclerosis type**			
Relapsing-remitting MS (RRMS)	203 (72.2)	170 (83.7)	33 (16.3)
Primary progressive MS (PPMS)	26 (9.3)	19 (73.1)	7 (26.9)
Secondary progressive MS (SPMS)	30 (10.7)	26 (86.7)	4 (13.3)
Other/Don’t know	22 (7.8)	18 (81.8)	4 (18.2)
**Time since diagnosis**			
<1 year	10 (3.6)	9 (90.0)	1 (10.0)
1–5 years	78 (27.8)	56 (71.8)	22 (28.2)
5.1–10 years	58 (20.6)	47 (81.0)	11 (19.0)
>10 years	135 (48.0)	121 (89.6)	14 (10.4)
**Current MS treatment**			
Tablets	106 (37.7)	94 (88.7)	12 (11.3)
Injectables	26 (9.3)	22 (84.6)	4 (15.4)
Intravenous	92 (32.7)	71 (77.2)	21 (22.8)
No specific treatment/Other	57 (20.3)	46 (80.7)	11 (19.3)
**MS control over the past 6 months**			
Yes	226 (80.4)	191 (84.5)	35 (15.5)
No	27 (9.6)	19 (70.4)	8 (29.6)
Don’t know	28 (10.0)	23 (82.1)	5 (17.9)
**No. of times missed disease modifying therapies in the past month**			
All of the time	7 (2.5)	4 (57.1)	3 (42.9)
Most of the time	3 (1.1)	3 (100.0)	0 (0.0)
Some of the time	9 (3.3)	6 (66.7)	3 (33.3)
Occasionally	36 (13.1)	32 (88.9)	4 (11.1)
Never	220 (80.0)	184 (83.6)	36 (16.4)
**MS affect daily activities in last 4 weeks**			
All of the time	44 (15.7)	33 (75.0)	11 (25.0)
Most of the time	35 (12.5)	31 (88.6)	4 (11.4)
Some of the time	85 (30.2)	69 (81.2)	16 (18.8)
Not very often	53 (18.9)	45 (84.9)	8 (15.1)
Not at all	64 (22.8)	55 (85.9)	9 (14.1)

* there was also “non-binary/prefer not to say”: 3 (1.1%) ** Other: 1 (0.4%). There was n = 1 (0.4%) who identified as Aboriginal/Torres Strait Islander. Abbreviations: AUD, Australian Dollars; K, 1000; RRMS, Relapsing-remitting MS; PPMS, Primary progressive MS; SPMS, Secondary progressive MS.

**Table 2:** Logistic regression predicting vaccine uptake using socio-demographic and clinical factors.

Category (Reference, n)	OR (95% CI)	*p*-Value
**Age (n = 281)**	1.06 (1.03–1.08)	<0.001
**Time since diagnosis (>10 years, n = 281)**		
<1 year	1.18 (0.14–10.22)	0.88
1–5 years	0.31 (0.15–0.66)	0.002
5.1–10 years	0.52 (0.22–1.24)	0.14

Regression analyses was controlled for time since study commencement. Abbreviations: OR (95% CI), Odds ratio (95% confidence interval).

**Table 3:** Linear regression predicting the Oxford COVID-19 Vaccine Hesitancy Scale score using socio-demographic and clinical factors.

	Step 1	Step 2			
Category (Reference, n)	Adj. R^2^	Adj. R^2^	Δ Adj. R^2^	B (SE)	*p*-Value
**Gender (Male, n = 261)**	0.043	0.068	0.025		
Female				2.21 (0.77)	0.005
**Age (n = 264)**	0.043	0.063	0.020	−0.07 (0.03)	0.01
**Location (Metropolitan, n = 264)**	0.043	0.043	0.000		
Regional/rural				3.82 (3.87)	0.32
**Time since diagnosis (>10 years, n = 264)**	0.043	0.050	0.007		
<1 year				−0.21 (1.84)	0.91
1–5 years				1.69 (0.79)	0.03
5.1–10 years				0.83 (0.86)	0.34

Step 1, time since study commencement is the only predictor variable entered into the model; step 2, the socio-demographic/clinical factor is the predictor variable entered into the model. Abbreviations: Adj. R^2^, Adjusted R^2^; B(SE), unstandardized coefficient (standard error).

## Data Availability

The data presented in this study are available on reasonable request from the corresponding author.

## Supplementary Materials

The following supporting information can be downloaded at: https://www.mdpi.com/article/10.3390/vaccines11020410/s1, List of MSVACCS investigators; Table S1. Survey items; Table S2. Hierarchical multivariable logistic regression analysis of vaccinated status; Table S3. The relationship between vaccinated status with intent to get vaccinated, belief about contracting COVID-19 within the next 12 months, and the scale and subscale indices; Table S4. Linear regression predicting the Oxford COVID-19 Confidence and Complacency Scale—summary and subscales’ scores using socio-demographic and clinical factors; Table S5. Hierarchical multivariable regression analysis of the Oxford COVID-19 Vaccine Hesitancy Scale score; Table S6. Hierarchical multivariable regression predicting the Oxford COVID-19 Confidence and Complacency Scale score; Table S7. Hierarchical multivariable regression predicting the Oxford COVID-19 Confidence and Complacency Scale—Beliefs about COVID-19 and Vaccine subscale score; Table S8. Hierarchical multivariable regression predicting the Oxford COVID-19 Confidence and Complacency Scale – Side-Effects subscale score; Table S9. Linear regression predicting the DIVAS-6 summary scale and subscales’ scores using socio-demographic and clinical factors; Table S10. Hierarchical multivariable regression predicting the DIVAS-6 Summary score; Table S11. Hierarchical multivariable regression predicting the DIVAS-6 Vaccine Vulnerability subscale score with time since diagnosis and MS control, controlling for gender and age; Table S12. Hierarchical multivariable regression predicting the DIVAS-6 Vaccine Vulnerability subscale score with MS impact on daily activities in the past four weeks, controlling for gender and age; Table S13. Receiver-operating analysis, showing the discriminative ability of each scale and subscale to predict vaccination status; Table S14. Sensitivity and specificity results for different cut off scores for the Oxford COVID-19 Vaccine Hesitancy Scale summary score; Table S15. Sensitivity and specificity results for different cut off scores for the Oxford COVID-19 Vaccine Confidence and Complacency Scale summary score; Table S16. Sensitivity and specificity results for different cut off scores for the Oxford COVID-19 Vaccine Confidence and Complacency Scale—Collective Importance subscale score; Table S17. Sensitivity and specificity results for different cut off scores for the Oxford COVID-19 Vaccine Confidence and Complacency Scale—Beliefs about COVID-19 subscale score; Table S18. Sensitivity and specificity results for different cut off scores for the Oxford COVID-19 Vaccine Confidence and Complacency Scale—Speed of Vaccine Development subscale score; Table S19. Sensitivity and specificity results for different cut off scores for the Oxford COVID-19 Vaccine Confidence and Complacency Scale—Side-Effects subscale score; Table S20. Sensitivity and specificity results for different cut off scores for the DIVAS-6—Disease Complacency subscale score; Table S21. Sensitivity and specificity results for different cut off scores for the DIVAS-6—Vaccine Vulnerability subscale score; Table S22. Sensitivity and specificity results for different cut off scores for the DIVAS-6 Summary Score; Table S23. Correlation matrix between significant variables from the univariable regression analyses, using Spearman’s Rho.
