# Supplementary material for: COVID-19 Vaccine Status, Intent, Hesitancy, and Disease-Related Beliefs in People with Multiple Sclerosis
Source: Vaccines (Basel). 2023 Feb 10;11(2):410. doi: 10.3390/vaccines11020410 (PMC9964563; doi:10.3390/vaccines11020410)
Supplement: Supplementary file 1 [file vaccines-11-00410-s001.zip › vaccines-2182516-supplementary.pdf]

Supplementary materials

These are supplementary materials to:

COVID-19 vaccine status, intent, hesitancy, and disease-related beliefs in people with multiple sclerosis.

Multiple Sclerosis patients' perspectives on coronavirus VACCination Survey (MSVACCS) investigators:

Monash Health, Victoria: Ms. Michelle Allan, Dr. Nathan Bain, Prof. Ernest Butler, Dr. Daphne Day, Dr. Lisa Grech, Dr. Amelia McCartney, Dr. Mike Nguyen, Prof. Eva Segelov, Dr. Kate Webber.

Sunshine Coast Hospital and Health Service, Queensland: Dr. Joshua Barton, Dr. Antony Winkel.

Table S1. Survey items

**Screening items**	
Are you 18 years or older?	Yes<br>No (Terminate if No)
Have you got MS?	Yes<br>No (Terminate if No)
Are you a [participating site] patient?	Yes<br>No (Terminate if No)
**Vaccination status**	
Have you already received a COVID-19 vaccine?	Yes, 1 dose only<br>Yes, 2 doses<br>No
**Oxford COVID-19 Vaccine Hesitancy Scale**<br>Instructions: We would like to know your feelings and thoughts about the COVID-19 vaccine. Note: If you have already been vaccinated against COVID-19, please complete these questions in relation to a future COVID-19 vaccine dose/booster.	
Would you take a COVID-19 vaccine if offered?	Definitely/have taken<br>Probably<br>I may or may not<br>Probably not<br>Definitely not<br>Don't know
When a COVID-19 vaccine is available:	I will want to get it as soon as possible<br>I will take it when offered<br>I'm not sure what I will do<br>I will put off (delay) getting it<br>I will refuse to get it<br>Don't know
I would describe my attitude towards receiving a COVID-19 vaccine as:	Very keen<br>Pretty positive<br>Neutral<br>Quite uneasy<br>Against it<br>Don't know
If a COVID-19 vaccine was available in my local area, I would:	Get it as soon as possible<br>Get it when I have time<br>Delay getting it<br>Avoid getting it for as long as possible<br>Never get it<br>Don't know
If my family or friends were thinking of getting a COVID-19 vaccination, I would:	Strongly encourage them<br>Encourage them<br>Not say anything to them about it<br>Ask them to delay getting the vaccination<br>Suggest that they do not get the vaccination<br>Don't know
I would describe myself as:	Eager to get a COVID-19 vaccine<br>Willing to get the COVID-19 vaccine<br>Not bothered about getting the COVID-19 vaccine<br>Unwilling to get the COVID-19 vaccine<br>Anti-vaccination for COVID-19<br>Don't know
Taking a COVID-19 vaccination is:	Really important<br>Important<br>Neither important nor unimportant<br>Unimportant<br>Really unimportant<br>Don't know
**Oxford COVID-19 Vaccine Confidence and Complacency Scale**	
Do you think you will be infected with COVID-19 over the next 12 months?	Definitely<br>Probably<br>Possibly<br>Probably not<br>Definitely not<br>Don't know

I think the COVID-19 vaccine is likely to:	Work for almost everyone<br>Work for most people<br>I am unsure how many people it will work for<br>Not work for most people<br>Not work for anyone<br>Don’t know
I think the COVID-19 vaccine is likely to:	Definitely work for me<br>Probably work for me<br>May or may not work for me<br>Probably not work for me<br>Definitely not work for me<br>Don’t know
I think if I get the COVID-19 vaccine it will be:	Really helpful for the community around me<br>Helpful for the community around me<br>Neither helpful nor unhelpful for the community around me<br>Unhelpful for the community around me<br>Really unhelpful for the community around me<br>Don’t know
I think if individuals like me get the COVID-19 vaccine it will:	Save a large number of lives<br>Save some lives<br>Have no impact<br>Lead to more deaths<br>Lead to a large number of deaths<br>Don’t know
I think the speed of developing and testing the vaccine means it will be:	Really good<br>Good<br>Will not affect how good or bad it is<br>Bad<br>Really bad<br>Don’t know
I think the speed of developing and testing the vaccine means it will be:	Really safe<br>Safe<br>It will not affect how safe it is<br>Unsafe<br>Really unsafe<br>Don’t know
I think if many people do not get the vaccine this:	Will be dangerous<br>May be dangerous<br>Will have no consequences at all<br>May be good<br>Will be good<br>Don’t know
I expect that receiving the vaccine will be:	Hardly noticeable<br>A little unpleasant<br>Moderately unpleasant<br>Painful<br>Extremely painful<br>Don’t know
I think the side-effects for people of getting the COVID-19 vaccine will be:	None<br>Mild<br>Moderate<br>Significant<br>Life-threatening<br>Don’t know
I think the COVID-19 vaccine will:	Greatly strengthen my immune system<br>Strengthen my immune system<br>It will neither strengthen nor weaken my immune system<br>Weaken my immune system<br>Greatly weaken my immune system<br>Don’t know
I think taking the COVID-19 vaccine:	Will give me complete freedom to get on with life just as before<br>Will give me greater freedom<br>Will have no effect on my freedom<br>Will restrict my freedom<br>Will completely restrict my freedom to get on with life

	Don't know
I think getting the vaccine is a sign of:	Great personal strength<br>Personal strength<br>Not a sign of personal strength or weakness<br>Personal weakness<br>Great personal weakness<br>Don't know
Taking a new COVID-19 vaccine will make me feel like a guinea pig:	Do not agree<br>Agree a little<br>Agree moderately<br>Agree a lot<br>Completely agree<br>Don't know
**Disease Influenced Vaccine Acceptance Scale-Six**<br>Instructions: We would like to know about how your MS may be related to your feelings and thoughts about the COVID-19 vaccine. For each of the following statements, please tap/click the one choice that best represents how strongly you agree or disagree with it. There are 6 choices to choose from for each statement.	
My history of MS makes me more worried about being infected with COVID-19:	Strongly agree<br>Somewhat agree<br>Neither disagree nor agree<br>Somewhat disagree<br>Strongly disagree<br>Don't know
My history of MS means having the vaccine is more important to me:	Strongly agree<br>Somewhat agree<br>Neither disagree nor agree<br>Somewhat disagree<br>Strongly disagree<br>Don't know
My doctor's recommendation regarding the vaccine is important to me:	Strongly agree<br>Somewhat agree<br>Neither disagree nor agree<br>Somewhat disagree<br>Strongly disagree<br>Don't know
My history of MS makes me worried about how well the vaccine will work for me:	Strongly disagree<br>Somewhat disagree<br>Neither disagree nor agree<br>Somewhat agree<br>Strongly agree<br>Don't know
My history of MS makes me worried about how the vaccine will affect me:	Strongly disagree<br>Somewhat disagree<br>Neither disagree nor agree<br>Somewhat agree<br>Strongly agree<br>Don't know
I am worried about how the vaccine will affect my MS treatment:	Strongly disagree<br>Somewhat disagree<br>Neither disagree nor agree<br>Somewhat agree<br>Strongly agree<br>Don't know
**Clinical**	
What type of MS do you have?	Relapsing-remitting MS (RRMS)<br>Primary progressive MS (PPMS)<br>Secondary progressive MS (SPMS)<br>Other (please specify): ____________<br>Don't know
How long have you had MS?	Less than 1 year<br>1 to 5 years<br>5.1 to 10 years<br>More than 10 years

My current treatment for my MS is/are (select all that apply):	Tablets<br>Injectables<br>Intravenous<br>No specific treatment<br>Other: ____
Over the past 6 months, is your MS well controlled?	Yes<br>No<br>Don't know
People often have difficulty taking their medications for one reason or another. How many times have you missed taking your disease modifying therapies (DMT) in the past month?	All of the time<br>Most of the time<br>Some of the time<br>Occasionally<br>Never
In the last four weeks, how much did your MS affect your daily activities?	All the time<br>Most of the time<br>Some of the time<br>Not very often<br>Not at all
**Socio-demographics**	
What is your gender?	Male<br>Female<br>Non-binary / third gender<br>Prefer not to say
What is your age?	[Text entry]: ____
What is your highest educational level (completed)?	No formal education<br>Primary education<br>Secondary education<br>Vocational/trade qualification<br>University education or higher degree<br>Other (please specify): ______________________
What is your annual household income (including everyone who lives in your home)?	Less than $50,000<br>$50,001 to $100,000<br>$100,001 to $150,000<br>More than $150,000<br>Prefer not to say
Do you identify as Aboriginal and/or Torres Strait Islander?	Yes<br>No<br>Prefer not to say
Is English your first language?	Yes<br>No
Please include any comments about your feelings and thoughts about your MS and COVID-19 vaccination that you would like to share. If you have no comments to include, please type 'Nil'.	[Text entry]: ____

Table S2. Hierarchical multivariable logistic regression analysis of vaccinated status (n = 281).

**Block and variable (reference)**	**B (SE)**	**OR (95% CI)**	**p-value**
Block 1			
**Time since study commencement**	-0.02 (0.02)	0.98 (0.95 – 1.01)	0.16
Block 2			
**Age**	0.05 (0.01)	1.06 (1.03 – 1.08)	<0.001
Block 3			
**Time since diagnosis (>10 years)**			
<1 year	1.16 (1.16)	3.18 (0.33 – 30.90)	0.32
1 – 5 years	-0.58 (0.43)	0.56 (0.24 – 1.30)	0.18
5.1 – 10 years	-0.38 (0.47)	0.69 (0.28 – 1.71)	0.42

Notes: Variables entered at each block: Block 1, time since study commencement; Block 2, age; Block 3, time since diagnosis. Abbreviations: B(SE), unstandardized coefficient (standard error); OR (95% CI), odds ratio (95% confidence interval).

Table S3. The relationship between vaccinated status with intent to get vaccinated, belief about contracting COVID-19 within the next 12 months, and the scale and subscale indices.

	MS - n, (%)		
	Vaccinated[a] (n = 233)	Unvaccinated (n = 48)	OR (95% CI)
**Intent to get vaccinated[b]**			
Likely (reference)	228 (91.6)	21 (8.4)	..
Unsure	5 (27.8)	13 (72.2)	0.04 (0.01 to 0.12)***
Unlikely	0 (0.0)	14 (100.0)	0 (..)
**Belief about contracting COVID-19 within the next 12 months[c]**			
Likely (reference)	124 (86.7)	19 (13.3)	..
Unlikely	70 (76.9)	21 (23.1)	0.49 (0.24 to 1.02)
Don't know	39 (83.0)	8 (17.0)	0.67 (0.26 to 1.74)
	**MS – M (SD)**		
	**Vaccinated[a] (n = 233)**	**Unvaccinated (n = 48)**	**OR (95% CI)**
Oxford COVID-19 Vaccine Hesitancy Summary Scale	9.1 (2.6)	19.0 (8.9)	0.68 (0.60 to 0.76)***
OCVCCS Summary	26.1 (4.1)	39.6 (12.7)	0.66 (0.52 to 0.83)***
OCVCCS: Collective Importance Subscale	7.9 (1.8)	12.5 (5.2)	0.56 (0.43 to 0.72)***
OCVCCS: Beliefs about COVID-19 Vaccine Subscale	7.1 (1.3)	9.1 (2.3)	0.49 (0.36 to 0.66)***
OCVCCS: Speed of Vaccine Development Subscale	6.4 (1.9)	10.0 (2.3)	0.37 (0.26 to 0.52)***
OCVCCS: Side-Effects Subscale	5.2 (1.5)	8.4 (2.9)	0.53 (0.43 to 0.65)***
DIVAS-6 Summary Scale	14.7 (3.5)	20.0 (4.7)	0.70 (0.62 to 0.80)***
DIVAS-6 Disease Complacency Subscale	5.8 (2.9)	7.6 (3.6)	0.82 (0.74 to 0.91)***
DIVAS-6 Vaccine Vulnerability Subscale	9.0 (3.2)	12.4 (2.7)	0.68 (0.58 to 0.79)***

*p<0.05; **p<.01; ***p<.001 for logistic regression models adjusted for covariates. Days since study commencement, age, and time since MS diagnosis were adjusted for all models.

[a] Vaccinated status was defined as receiving at least one COVID-19 vaccine dose.
[b] Vaccination intent were categorised as: Likely, Have or definitely/probably; Unsure, May or may not or possibly/don't know; Unlikely, Probably not or definitely not.
[c] Belief about contracting COVID-19 within the next 12 months were categorised as: Likely, Definitely or probably/possibly; Unlikely, Probably not or Definitely Not; Don't know.

Abbreviations: MS, multiple sclerosis; OR, odds ratio; OCVCCS, Oxford COVID-19 Vaccine Confidence and Complacency Scale; DIVAS-6, Disease Influenced Vaccine Acceptance Scale-6; 95% CI, 95% confidence interval.

Table S4. Linear regression predicting the Oxford COVID-19 Confidence and Complacency Scale – summary and subscales' scores using socio-demographic and clinical factors.

	Step 1	Step 2			
**Category (reference, n)**	**Adj. R^2**	**Adj. R^2**	**Δ Adj. R^2**	**B (SE)**	**p-value**
Summary scale score					
**Age (n = 163)**	0.005	0.049	0.044	-0.13 (0.04)	0.004
**Location (Metropolitan, n = 163)**	0.005	0.036	0.031		
Regional/rural				16.29 (6.54)	0.01
**Time since diagnosis (>10 years, n =163)**	0.005	0.068	0.063		
<1 year				0.11 (2.71)	0.97
1 – 5 years				5.08 (1.40)	<0.001
5.1 – 10 years				1.39 (1.48)	0.35
**MS control over the past 6 months (Yes, n = 163)**	0.005	0.018	0.013		
No				4.31 (2.18)	0.05
Don't know				1.16 (1.94)	0.55
Beliefs about COVID-19 and Vaccine subscale score					
**Age (n = 228)**	-0.002	0.012	0.014	-0.02 (0.01)	0.04
**Time since diagnosis (>10 years, n = 228)**	-0.002	0.037	0.039		
<1 year				-0.26 (0.56)	0.64
1 – 5 years				0.77 (0.27)	0.004
5.1 – 10 years				0.70 (0.29)	0.02
Side-Effects subscale score					
**Age (n = 247)**	0.002	0.032	0.030	-0.03 (0.01)	0.004
**Time since diagnosis (>10 years, n = 247)**	0.002	0.029	0.027		
<1 year				-0.51 (0.74)	0.49
1 – 5 years				0.92 (0.33)	0.01
5.1 – 10 years				0.40 (0.35)	0.25
**MS control over the past 6 months (Yes, n = 247)**	0.002	0.012	0.010		
No				0.88 (0.50)	0.08
Don't know				0.55 (0.45)	0.22
Collective Importance subscale score					
**Location (Metropolitan, n = 226)**	0.005	0.037	0.032		
Regional/rural				6.39 (2.22)	<0.001
**Current MS treatment (Yes, n = 226)**	0.005	0.003	-0.002		
No				0.31 (0.48)	0.52
**MS control over the past 6 months (Yes, n = 226)**	0.005	0.007	0.002		
No				1.08 (0.69)	0.12
Don't know				0.04 (0.64)	0.95
Speed of Vaccine Development subscale score					
**Age (n = 222)**	0.028	0.038	0.010	-0.02 (0.01)	0.08
**Location (Metropolitan, n = 222)**	0.028	0.049	0.021		
Regional/rural				4.51 (1.86)	0.02

Notes: Step 1, time since study commencement is the only predictor variable entered into the model; Step 2, the socio-demographic/clinical factor is the predictor variable entered into the model. Abbreviations: Adj. R^2, Adjusted R^2; B(SE), unstandardized coefficient (standard error).

Table S5. Hierarchical multivariable regression analysis of the Oxford COVID-19 Vaccine Hesitancy Scale score (n = 261).

**Step and variable (reference)**	**Adj. R^2**	**ΔAdj. R^2**	**B (SE)**	**p-value**	**sr**
Step 1	0.043	0.043			
**Time since study commencement**			0.12 (0.04)	<0.001	0.22
Step 2	0.086	0.043			
**Gender (Male)**					
Female			2.00 (0.77)	0.01	0.16
**Age**			-0.06 (0.03)	0.01	-0.15
Step 3	0.085	-0.001			
**Time since diagnosis (>10 years)**					
<1 year			-1.26 (1.94)	0.52	-0.04
1 – 5 years			1.00 (0.87)	0.25	0.07
5.1 – 10 years			0.56 (0.86)	0.52	0.04

Notes: Variables entered into the model at each step: Step 1, time since study commencement; Step 2, gender and age; Step 3, time since diagnosis. Variable categories excluded: Other/Don't Know (gender). Abbreviations: Adj. R^2, Adjusted R^2; B(SE), unstandardized coefficient (standard error); sr, semipartial correlation coefficient.

Table S6. Hierarchical multivariable regression predicting the Oxford COVID-19 Confidence and Complacency Scale score (n = 163).

Step and variable (reference)	Adj. R^2	ΔAdj. R^2	B (SE)	p-value	sr
Step 1	0.005	0.005			
**Time since study commencement**			0.09 (0.06)	0.18	0.11
Step 2	0.069	0.064			
**Age**			-0.11 (0.05)	0.01	-0.20
**Location (Metropolitan)**					
Regional/rural			13.56 (6.51)	0.04	0.16
Step 3	0.102	0.033			
**Time since diagnosis (>10 years)**					
<1 year			-2.23 (2.80)	0.43	-0.06
1 – 5 years			3.65 (1.47)	0.01	0.19
5.1 – 10 years			0.82 (1.47)	0.58	0.04

Notes: Variables entered into the model at each step: Step 1, time since study commencement; Step 2, age and location; Step 3, time since diagnosis. Abbreviations: Adj. R^2, Adjusted R^2; B(SE), unstandardized coefficient (standard error); sr, semipartial correlation coefficient.

Table S7. Hierarchical multivariable regression predicting the Oxford COVID-19 Confidence and Complacency Scale – Beliefs about COVID-19 and Vaccine subscale score (n = 228).

Step and variable (reference)	Adj. R^2	ΔAdj. R^2	B (SE)	p-value	sr
Step 1	-0.002	-0.002			
**Time since study commencement**			0.01 (0.01)	0.44	0.05
Step 2	0.012	0.014			
**Age**			-0.02 (0.01)	0.04	-0.14
Step 3	0.041	0.029			
**Time since diagnosis (>10 years)**					
<1 year			-0.49 (0.58)	0.40	-0.06
1 – 5 years			0.62 (0.29)	0.03	0.14
5.1 – 10 years			0.63 (0.30)	0.03	0.14

Notes: Variables entered into the model at each step: Step 1, time since study commencement; Step 2, age; Step 3, time since diagnosis. Abbreviations: Adj. R^2, Adjusted R^2; B(SE), unstandardized coefficient (standard error); sr, semipartial correlation coefficient.

Table S8. Hierarchical multivariable regression predicting the Oxford COVID-19 Confidence and Complacency Scale – Side-Effects subscale score (n = 247).

Step and variable (reference)	Adj. R^2	ΔAdj. R^2	B (SE)	p-value	sr
Step 1	0.002	0.002			
**Time since study commencement**			0.02 (0.02)	0.21	0.08
Step 2	0.032	0.030			
**Age**			-0.03 (0.01)	0.004	-0.18
Step 3	0.046	0.016			
**Time since diagnosis (>10 years)**					
<1 year			-1.01 (0.76)	0.18	-0.08
1 – 5 years			0.61 (0.35)	0.08	0.11
5.1 – 10 years			0.27 (0.35)	0.45	0.05

Notes: Variables entered into the model at each step: Step 1, time since study commencement; Step 2, age; Step 3, time since diagnosis. Abbreviations: Adj. R^2, Adjusted R^2; B(SE), unstandardized coefficient (standard error); sr, semipartial correlation coefficient.

Table S9. Linear regression predicting the DIVAS-6 summary scale and subscales' scores using socio-demographic and clinical factors.

	Step 1	Step 2			
**Category (reference, n)**	**Adj. R^2**	**Adj. R^2**	**Δ Adj. R^2**	**B (SE)**	**p-value**
Summary scale score					
**Age (n = 254)**	0.008	0.020	0.012	-0.04 (0.02)	0.04
**English as first language (Yes, n = 254)**	0.008	0.037	0.029		
No				2.68 (0.92)	0.004
**Current MS treatment (Yes, n = 254)**	0.008	0.006	-0.002		
No				0.47 (0.66)	0.47
Vaccine Vulnerability subscale score					
**Gender (Male, n = 259)**	0.006	0.026	0.020		
Female				1.20 (0.48)	0.01
**Age (n = 262)**	0.006	0.037	0.031	-0.05 (0.02)	0.002
**Time since diagnosis (>10 years, n = 262)**	0.006	0.032	0.026		
<1 year				-0.78 (1.21)	0.52
1 – 5 years				1.36 (0.50)	0.01
5.1 – 10 years				1.07 (0.54)	0.051
**MS control over the past 6 months (Yes, n = 262)**	0.006	0.022	0.016		
No				1.67 (0.69)	0.02
Don't know				0.75 (0.72)	0.30
**MS impact on daily activities in the past four weeks (Not at all, n = 262)**	0.006	0.063	0.057		
All of the time				2.00 (0.66)	<0.001
Most of the time				1.04 (0.70)	0.14
Some of the time				2.19 (0.56)	<0.001
Not very often				2.16 (0.64)	<0.001
Disease Complacency subscale score					
**MS Type (Relapsing-remitting MS, n = 247)**	0.00	0.020	0.020		
Primary progressive MS				1.53 (0.64)	0.02
Secondary progressive MS				0.87 (0.61)	0.15

Notes: Step 1, time since study commencement is the only predictor variable entered into the model; Step 2, the socio-demographic/clinical factor is the predictor variable entered into the model. These variables were excluded due to <5 responses: non-binary/other gender. Abbreviations: Adj. R^2, Adjusted R^2; B(SE), unstandardized coefficient (standard error).

Table S10. Hierarchical multivariable regression predicting the DIVAS-6 Summary score (n = 247).

**Step and variable (reference)**	**Adj. R^2**	**ΔAdj. R^2**	**B (SE)**	**p-value**	**sr**
Step 1	0.008	0.008			
**Time since study commencement**			0.05 (0.03)	0.08	0.11
Step 2	0.046	0.038			
**Age**			-0.04 (0.02)	0.06	-0.12
**English as first language (Yes)**					
No			2.56 (0.92)	0.01	0.17

Notes: Variables entered into the model at each step: Step 1, time since study commencement; Step 2, age and English as first language. Adj. R^2, Adjusted R^2; B(SE), unstandardized coefficient (standard error); sr, semipartial correlation coefficient.

Table S11. Hierarchical multivariable regression predicting the DIVAS-6 Vaccine Vulnerability subscale score with time since diagnosis and MS control, controlling for gender and age (n = 259).

Step and variable (reference)	Adj. R^2	ΔAdj. R^2	B (SE)	p-value	sr
Step 1	0.006	0.006			
**Time since study commencement**			0.04 (0.02)	0.11	0.10
Step 2	0.058	0.052			
**Gender (Male)**					
Female			1.04 (0.48)	0.03	0.13
**Age**			-0.05 (0.02)	0.002	-0.19
Step 3	0.094	0.036			
**Time since diagnosis (>10 years)**					
<1 year			-1.67 (1.21)	0.17	-0.08
1 – 5 years			0.87 (0.53)	0.10	0.10
5.1 – 10 years			0.90 (0.54)	0.09	0.10
**MS control over the past 6 months (Yes)**					
No			1.77 (0.67)	0.01	0.16
Don't know			1.04 (0.71)	0.14	0.09

Notes: Variables entered into the model at each step: Step 1, time since study commencement; Step 2, gender and age; Step 3, time since diagnosis and MS control over the past 6 months. Variable categories excluded: Other/Don't Know (gender). Adj. R^2, Adjusted R^2; B(SE), unstandardized coefficient (standard error); sr, semipartial correlation coefficient.

Table S12. Hierarchical multivariable regression predicting the DIVAS-6 Vaccine Vulnerability subscale score with MS impact on daily activities in the past four weeks, controlling for gender and age (n = 259).

Step and variable (reference)	Adj. R^2	ΔAdj. R^2	B (SE)	p-value	sr
Step 1	0.006	0.006			
**Time since study commencement**			0.04 (0.02)	0.11	0.10
Step 2	0.058	0.052			
**Gender (Male)**					
Female			1.04 (0.48)	0.03	0.13
**Age**			-0.05 (0.02)	0.002	-0.19
Step 3	0.134	0.076			
**MS impact on daily activities in the past four weeks (Not at all)**					
All of the time			2.54 (0.65)	<0.001	0.23
Most of the time			2.28 (0.71)	0.002	0.19
Some of the time			2.40 (0.53)	<0.001	0.26
Not very often			2.28 (0.61)	<0.001	0.22

Notes: Variables entered into the model at each step: Step 1, time since study commencement; Step 2, gender and age; Step 3, MS impact on daily activities in the past four weeks. Variable categories excluded: Other/Don't Know (gender). Adj. R^2, Adjusted R^2; B(SE), unstandardized coefficient (standard error); sr, semipartial correlation coefficient.

Table S13. Receiver-operating analysis, showing the discriminative ability of each scale and subscale to predict vaccination status.

**Scale**	**Area under the curve**	**SE**	**p-value**	**95% CI Lower**	**95% CI Upper**
Oxford COVID-19 Vaccine Hesitancy Scale	0.87	0.04	<0.001	0.80	0.94
OCVCCS Summary	0.89	0.03	<0.001	0.83	0.96
OCVCCS Collective Importance Subscale	0.81	0.05	<0.001	0.71	0.90
OCVCCS Beliefs about COVID-19 Vaccine Subscale	0.77	0.05	<0.001	0.69	0.86
OCVCCS Speed of Vaccine Development Subscale	0.88	0.03	<0.001	0.82	0.94
OCVCCS Side-Effects Subscale	0.85	0.04	<0.001	0.78	0.92
DIVAS-6 Disease Complacency	0.66	0.05	<0.001	0.57	0.75
DIVAS-6 Vaccine Vulnerability	0.80	0.04	<0.001	0.73	0.88
DIVAS-6 Summary	0.81	0.04	<0.001	0.74	0.89

Notes: OCVCCS, Oxford COVID-19 Vaccine Confidence and Complacency Scale; DIVAS-6, Disease Influenced COVID-19 Vaccine Acceptance Scale-Six; SE, Standard error.

Table S14. Sensitivity and specificity results for different cut off scores for the Oxford COVID-19 Vaccine Hesitancy Scale summary score.

Cut-off Scores ≥	Sensitivity	Specificity	Youden's Index
6	1.00	0.00	0.00
8	0.95	0.43	0.38
9	0.87	0.57	0.43
10	0.87	0.67	0.54
11	0.82	0.75	0.57
12	0.74	0.84	0.58
13	0.71	0.88	0.59
14	0.66	0.92	0.57
15	0.61	0.94	0.54
16	0.55	0.98	0.53
17	0.50	0.99	0.49
18	0.45	0.99	0.44
19	0.42	1.00	0.42
21	0.40	1.00	0.40
23	0.37	1.00	0.37
24	0.34	1.00	0.34
25	0.32	1.00	0.32
26	0.26	1.00	0.26
27	0.24	1.00	0.24
28	0.21	1.00	0.21
29	0.18	1.00	0.18
31	0.16	1.00	0.16
34	0.05	1.00	0.05
35	0.03	1.00	0.03
36	0.00	1.00	0.00

Table S15. Sensitivity and specificity results for different cut off scores for the Oxford COVID-19 Vaccine Confidence and Complacency Scale summary score.

**Cut-off Scores ≥**	**Sensitivity**	**Specificity**	**Youden's Index**
14	1.00	0.00	0.00
17	1.00	0.01	0.01
19	1.00	0.02	0.02
20	1.00	0.06	0.06
21	1.00	0.12	0.12
22	1.00	0.15	0.15
23	1.00	0.18	0.18
24	1.00	0.25	0.25
25	1.00	0.34	0.34
26	1.00	0.39	0.39
27	1.00	0.54	0.54
28	1.00	0.64	0.64
29	0.80	0.70	0.50
30	0.70	0.78	0.48
31	0.70	0.85	0.55
32	0.65	0.92	0.57
33	0.55	0.95	0.50
35	0.50	0.99	0.49
37	0.45	0.99	0.44
38	0.35	0.99	0.34
39	0.35	1.00	0.35
45	0.30	1.00	0.30
52	0.25	1.00	0.25
55	0.20	1.00	0.20
57	0.15	1.00	0.15
59	0.10	1.00	0.10
64	0.05	1.00	0.05
68	0.00	1.00	0.00

Table S16. Sensitivity and specificity results for different cut off scores for the Oxford COVID-19 Vaccine Confidence and Complacency Scale – Collective Importance subscale score.

Cut-off Scores ≥	Sensitivity	Specificity	Youden's Index
4	1.00	0.00	0.00
6	1.00	0.08	0.08
7	0.97	0.22	0.18
8	0.86	0.47	0.33
9	0.76	0.68	0.43
10	0.66	0.83	0.48
11	0.48	0.93	0.42
12	0.45	0.98	0.42
13	0.35	0.98	0.32
14	0.31	1.00	0.31
16	0.28	1.00	0.28
18	0.24	1.00	0.24
20	0.17	1.00	0.17
21	0.10	1.00	0.10
23	0.03	1.00	0.03
25	0.00	1.00	0.00

Table S17. Sensitivity and specificity results for different cut off scores for the Oxford COVID-19 Vaccine Confidence and Complacency Scale – Beliefs about COVID-19 subscale score.

Cut-off Scores ≥	Sensitivity	Specificity	Youden's Index
2	1.00	0.00	0.00
4	1.00	0.02	0.02
5	1.00	0.02	0.02
6	1.00	0.09	0.09
7	0.92	0.32	0.24
8	0.73	0.64	0.37
9	0.51	0.90	0.41
10	0.35	0.96	0.31
11	0.19	1.00	0.19
12	0.16	1.00	0.16
13	0.11	1.00	0.11
14	0.08	1.00	0.08
15	0.03	1.00	0.03
16	0.00	1.00	0.00

Table S18. Sensitivity and specificity results for different cut off scores for the Oxford COVID-19 Vaccine Confidence and Complacency Scale – Speed of Vaccine Development subscale score.

Cut-off Scores ≥	Sensitivity	Specificity	Youden's Index
2	1.00	0.00	0.00
4	1.00	0.11	0.11
5	1.00	0.17	0.17
6	1.00	0.31	0.31
7	0.97	0.52	0.50
8	0.76	0.74	0.49
9	0.70	0.84	0.54
10	0.57	0.98	0.55
11	0.51	0.98	0.50
12	0.24	1.00	0.24
13	0.16	1.00	0.16
14	0.03	1.00	0.03
15	0.00	1.00	0.00

Table S19. Sensitivity and specificity results for different cut off scores for the Oxford COVID-19 Vaccine Confidence and Complacency Scale – Side-Effects subscale score.

Cut-off Scores ≥	Sensitivity	Specificity	Youden's Index
2	1.00	0.00	0.00
4	1.00	0.03	0.03
5	0.95	0.36	0.31
6	0.87	0.68	0.55
7	0.71	0.85	0.56
8	0.50	0.92	0.42
9	0.37	0.95	0.32
10	0.29	0.98	0.27
11	0.24	1.00	0.23
13	0.13	1.00	0.13
14	0.05	1.00	0.05
15	0.03	1.00	0.03
16	0.00	1.00	0.00

Table S20. Sensitivity and specificity results for different cut off scores for the DIVAS-6 – Disease Complacency subscale score.

Cut-off Scores ≥	Sensitivity	Specificity	Youden's Index
2	1.00	0.00	0.00
4	0.82	0.28	0.10
5	0.80	0.41	0.21
6	0.73	0.58	0.31
7	0.57	0.68	0.25
8	0.43	0.79	0.22
9	0.27	0.85	0.12
10	0.27	0.88	0.16
11	0.23	0.91	0.13
12	0.21	0.95	0.16
13	0.14	0.96	0.10
14	0.07	0.98	0.05
15	0.05	0.98	0.02
16	0.00	1.00	0.00

Table S21. Sensitivity and specificity results for different cut off scores for the DIVAS-6 – Vaccine Vulnerability subscale score.

Cut-off Scores ≥	Sensitivity	Specificity	Youden's Index
2	1.00	0.00	0.00
4	0.98	0.08	0.05
5	0.98	0.13	0.11
6	0.98	0.17	0.14
7	0.96	0.22	0.17
8	0.96	0.30	0.26
9	0.91	0.37	0.28
10	0.86	0.56	0.42
11	0.84	0.67	0.52
12	0.73	0.77	0.50
13	0.55	0.87	0.42
14	0.39	0.93	0.31
15	0.30	0.95	0.25
16	0.00	1.00	0.00

Table S22. Sensitivity and specificity results for different cut off scores for the DIVAS-6 Summary Score.

Cut-off Scores ≥	Sensitivity	Specificity	Youden's Index
5	1.00	0.00	0.00
7	1.00	0.01	0.01
8	1.00	0.03	0.03
9	1.00	0.05	0.05
10	1.00	0.08	0.08
11	1.00	0.12	0.12
12	0.98	0.16	0.14
13	0.98	0.26	0.23
14	0.95	0.36	0.31
15	0.90	0.46	0.36
16	0.85	0.55	0.41
17	0.76	0.71	0.47
18	0.66	0.77	0.42
19	0.54	0.90	0.43
20	0.51	0.93	0.44
21	0.37	0.96	0.32
22	0.32	0.98	0.29
23	0.27	0.99	0.25
24	0.22	1.00	0.22
25	0.20	1.00	0.19
26	0.17	1.00	0.17
27	0.15	1.00	0.15
28	0.10	1.00	0.10
29	0.05	1.00	0.05
30	0.02	1.00	0.02
31	0.00	1.00	0.00

Table S23. Correlation matrix between significant variables from the univariable regression analyses, using Spearman's Rho.

		Age (Continuous)	Location	Gender	Annual Household Income	English as first language	MS Type	Time since MS diagnosis	MS control over past 6 months	MS affect daily activities in the past four weeks
Age (Continuous)	Correlation Coefficient	1.000	-.162**	-.151*	-.068	-.068	.322**	.437**	-.005	-.227**
	Sig. (2-tailed)	.	.006	.011	.254	.259	<0.001	<0.001	.930	<0.001
	N	281	281	281	281	281	281	281	281	281
Location	Correlation Coefficient	-.162**	1.000	.054	-.003	-.128*	-.056	-.260**	.071	-.024
	Sig. (2-tailed)	.006	.	.363	.954	.032	.348	<0.001	.232	.684
	N	281	281	281	281	281	281	281	281	281
Gender	Correlation Coefficient	-.151*	.054	1.000	.023	-.107	-.181**	-.092	.019	.115
	Sig. (2-tailed)	.011	.363	.	.703	.073	.002	.124	.745	.055
	N	281	281	281	281	281	281	281	281	281
Annual Household Income	Correlation Coefficient	-.068	-.003	.023	1.000	.017	.060	-.004	-.033	.100
	Sig. (2-tailed)	.254	.954	.703	.	.772	.316	.940	.582	.094
	N	281	281	281	281	281	281	281	281	281
English as first language	Correlation Coefficient	-.068	-.128*	-.107	.017	1.000	.091	-.098	.082	-.056
	Sig. (2-tailed)	.259	.032	.073	.772	.	.127	.101	.168	.347
	N	281	281	281	281	281	281	281	281	281
MS Type	Correlation Coefficient	.322**	-.056	-.181**	.060	.091	1.000	.116	.188**	-.331**
	Sig. (2-tailed)	<0.001	.348	.002	.316	.127	.	.052	.002	<0.001
	N	281	281	281	281	281	281	281	281	281
Time since MS diagnosis	Correlation Coefficient	.437**	-.260**	-.092	-.004	-.098	.116	1.000	-.087	-.070
	Sig. (2-tailed)	<0.001	<0.001	.124	.940	.101	.052	.	.146	.239
	N	281	281	281	281	281	281	281	281	281

**MS control over past 6 months**	**Correlation Coefficient**	-.005	.071	.019	-.033	.082	.188**	-.087	1.000	-.329**
	**Sig. (2-tailed)**	.930	.232	.745	.582	.168	.002	.146	.	<0.001
	**N**	281	281	281	281	281	281	281	281	281
**MS affect daily activities in the past four weeks**	**Correlation Coefficient**	-.227**	-.024	.115	.100	-.056	-.331**	-.070	-.329**	1.000
	**Sig. (2-tailed)**	<0.001	.684	.055	.094	.347	<0.001	.239	<0.001	.
	**N**	281	281	281	281	281	281	281	281	281

Notes: *p<0.05; **p<0.01.
